# Soil Temperature Determines the Reaction of Olive Cultivars to *Verticillium dahliae* Pathotypes

**DOI:** 10.1371/journal.pone.0110664

**Published:** 2014-10-17

**Authors:** Rocío Calderón, Carlos Lucena, José L. Trapero-Casas, Pablo J. Zarco-Tejada, Juan A. Navas-Cortés

**Affiliations:** Instituto de Agricultura Sostenible (IAS), Consejo Superior de Investigaciones Científicas (CSIC), Apartado 4084, Campus de Excelencia Internacional Agroalimentario, Córdoba, Spain; University of Nottingham, United Kingdom

## Abstract

**Background:**

Development of Verticillium wilt in olive, caused by the soil-borne fungus *Verticillium dahliae*, can be influenced by biotic and environmental factors. In this study we modeled i) the combined effects of biotic factors (i.e., pathotype virulence and cultivar susceptibility) and abiotic factors (i.e., soil temperature) on disease development and ii) the relationship between disease severity and several remote sensing parameters and plant stress indicators.

**Methodology:**

Plants of Arbequina and Picual olive cultivars inoculated with isolates of defoliating and non-defoliating *V. dahliae* pathotypes were grown in soil tanks with a range of soil temperatures from 16 to 32°C. Disease progression was correlated with plant stress parameters (i.e., leaf temperature, steady-state chlorophyll fluorescence, photochemical reflectance index, chlorophyll content, and ethylene production) and plant growth-related parameters (i.e., canopy length and dry weight).

**Findings:**

Disease development in plants infected with the defoliating pathotype was faster and more severe in Picual. Models estimated that infection with the defoliating pathotype was promoted by soil temperatures in a range of 16 to 24°C in cv. Picual and of 20 to 24°C in cv. Arbequina. In the non-defoliating pathotype, soil temperatures ranging from 16 to 20°C were estimated to be most favorable for infection. The relationship between stress-related parameters and disease severity determined by multinomial logistic regression and classification trees was able to detect the effects of *V. dahliae* infection and colonization on water flow that eventually cause water stress.

**Conclusions:**

Chlorophyll content, steady-state chlorophyll fluorescence, and leaf temperature were the best indicators for Verticillium wilt detection at early stages of disease development, while ethylene production and photochemical reflectance index were indicators for disease detection at advanced stages. These results provide a better understanding of the differential geographic distribution of *V. dahliae* pathotypes and to assess the potential effect of climate change on Verticillium wilt development.

## Introduction

Verticillium wilt (VW) of olive (*Olea europea* L.), caused by the fungus *Verticillium dahliae* Kleb., is the most important soil-borne disease affecting olive trees worldwide [1], [2] and can cause severe yield losses and plant death [3]. The disease was first observed in Italy in 1946 [4] and is now present in many Mediterranean countries and in California, USA [1]. In Spain, the spread of Verticillium wilt in olive trees has been associated with the expansion of olive cultivation and changes in cropping practices aimed at increasing yields [5]. Such changes include the use of self-rooted planting stocks to establish high-density plantings, drip irrigation, reduced or no tillage, and high inputs of fertilizer in newly cultivated soils or fertile soils [6] previously cropped with plants susceptible to *V. dahliae*, such as cotton [5].

Microsclerotia, the long-lasting surviving structures of *V. dahliae*, constitute the main potential infective inoculum of the pathogen in field soils, where it can survive for up to 15 years [7]. These structures germinate multiple times in response to root exudates [8] and favorable soil environmental conditions, forming hyphae that penetrate the plant root, grow across the root cortex, and upon reaching the xylem vessels facilitate the rapid upward spread of the pathogen by conidia transported in the transpiration stream [9]. As a result of xylem colonization by the pathogen, water flow decreases, leading to water stress [10]. Infection with *V. dahliae* in olive trees has resulted in two main disease syndromes -defoliating (D) and non-defoliating (ND), which are induced by specific D and ND *V. dahliae* pathotypes, respectively [1], [11]. The D syndrome is characterized by early drop of asymptomatic green leaves from individual twigs and branches, eventually leading to complete defoliation and necrosis [1]. These symptoms can develop from late fall to late spring [11]. Conversely, the ND syndrome comprises two symptom complexes: (i) apoplexy, a rapid and extensive dieback of twigs and branches of olive trees without loss of leaves occurring in late winter, and (ii) slow decline, mainly characterized by flower mummification and necrosis of inflorescences along with leaf chlorosis and necrosis [1], which occurs during springtime [11]. Infections with the D pathotype can be lethal to the plant, whereas ND-infected olive trees may eventually show remission from symptoms [3], [12]. D and ND *V. dahliae* pathotypes also have different modes of dispersal and produce different spatial patterns of disease [11]. Infections with the D pathotype are of greater concern because the pathogen can spread rapidly over short and relatively long distances through windblown infected leaves that fall early and in large numbers from diseased trees [11]. Planting resistant cultivars is the most effective measure for controlling and limiting the spread of Verticillium wilt [1]. However, the most widely used olive cultivars in Spain (i.e., Picual and Arbequina) have been found to be highly susceptible and susceptible to D *V. dahliae*, respectively, and susceptible and moderately resistant to ND *V. dahliae*, respectively, under controlled conditions in artificial inoculation tests [13].

A recent study on Verticillium wilt in olive trees has shown that overall disease incidence is related to initial inoculum density in the soil [14]. However, the development of symptoms in relation to inoculum density is variable and strongly influenced by environmental and soil conditions [15]. Soil temperature is a critical factor for the development of Verticillium wilt and fungal growth [16]. Using soil tanks, McKeen [17] found that infection of potato by *V. albo-atrum* occurred between 12 and 32°C, but symptom expression was greatest between 20 and 28°C. In southern Spain, Bejarano-Alcázar et al. [18] reported that the optimal temperature for in vitro growth of *V. dahliae* isolates from cotton ranged from 24 to 27°C in the D pathotype and from 21 to 24°C in the ND pathotype. In China, Xu et al. [19] determined that the optimal growth temperature for *V. dahliae* isolates of the D and ND pathotype was 25°C, although D pathotype isolates can adapt well to high temperatures and severely infect cotton at temperatures ranging from 25 to 30°C. In olive plants, development of Verticillium wilt is favored by air and soil temperatures close to the optimal growth range of *V. dahliae* [12]. In Mediterranean-type climates, severity of Verticillium wilt attacks is favored by moderate air temperatures during spring, but high summer temperatures suppress further development of the disease [3], [11]. Nevertheless, very little is known about the influence of the physical environment on Verticillium wilt in olive trees, which limits our understanding of the disease [1].

In plants, water stress caused either by *V. dahliae* infection or drought induces stomatal closure, which reduces the transpiration rate [10]. As a result, evaporative cooling decreases and leaf temperature increases. In the past, this increase in leaf temperature has been detected early using thermal infrared radiation [20] recorded by spectrometers at ground level. The visible part of the spectrum has also been used for early water stress detection based on indices that use bands at specific wavelengths in which photosynthetic pigments are affected by stress. An example of such indices is the photochemical reflectance index (PRI), a narrowband spectral index [21] that is sensitive to the epoxidation state of xanthophyll-cycle pigments and to photosynthetic efficiency, serving as a proxy for water stress detection at leaf level [22]. Another indicator of water stress is chlorophyll fluorescence emission, as shown by several laboratory studies that have found it to be strongly correlated with photosynthesis and other physiological processes [23]. Over the last ten years, scientific interest in steady-state chlorophyll fluorescence (Fs) (i.e., fluorescence emitted under constant illumination without saturation flashes) has increased because measurements of Fs do not require high-energy sources and can be conducted remotely using active or passive methods [24]. In particular, leaf-level Fs measurements obtained with instruments known as pulse amplitude modulating (PAM) fluorometers have been used successfully to detect plant water stress [24]. In fact, *Minolta Corporation* developed a portable chlorophyll meter (SPAD) in the 1990s to take rapid measurements of chlorophyll content in leaves. This instrument uses two light-emitting diodes (650 and 940 nm) and a photodiode detector to measure transmission of red and infrared light through plant leaves. Given that there is a close relationship between leaf chlorophyll content and the output of the SPAD meter [25], such measurements have been used to assess stress in crops [25], [26], revealing that water stress levels decrease chlorophyll content in leaves and consequently SPAD readings.

Leaf-level remote sensing studies have been conducted to detect and assess diseases in various crops. Most of these studies have focused on foliar pathogens in annual crops. Leaf temperature has been shown to successfully detect water stress caused by soil-borne pathogens, as mentioned above. In fact, Pinter et al. [27] recorded leaf temperatures 3–4°C higher than those of healthy plants in sugar beet and cotton crops affected by *Pythium aphanidermatum* and *Pymatotrichum omnivorum*, respectively. Other examples of leaf temperature measurements for the detection of root diseases include beans infected by *Fusarium solani*, *Pythium ultimum*, and *Rhizoctonia solani* [28], soybeans affected by brown stem rot caused by *Phialophora solani* [29], and wheat with moderate take-all symptoms caused by *Gaeumannomyces graminis* var. *tritici* [30]. As regards *V. dahliae* infection, Nilsson [31] reported that infected oilseed rape plants exhibited leaf temperatures 5–8°C higher than those of non-infected plants. Calderón et al. [32] found increases of 2°C at early stages of Verticillium wilt development in olive trees under field conditions. Chlorophyll fluorescence was also found to be a good indicator for detecting Verticillium wilt at early stages of the disease, while the photochemical reflectance and chlorophyll indices were good indicators for detecting the disease at advanced stages [32].

The objectives of this study were (i) to quantify the combined effects of biotic factors (i.e., pathotype virulence and cultivar susceptibility) and abiotic factors (i.e., soil temperature) on the development of Verticillium wilt and olive tree growth and (ii) to determine the relationship between stress indicators including several remote sensing parameters and VW severity according to the hypothesis that thermal, reflectance, and fluorescence measurements are sensitive to physiological changes induced by infection and colonization by *V. dahliae* pathotypes.

## Materials and Methods

### 1. *Verticillium dahliae* isolates, olive plants, inoculation, and growth conditions

Olive plants were inoculated with *V. dahliae* isolates V138 (D) and V176 (ND), which have been characterized in previous studies [33] and are deposited in the culture collection of the *Departamento de Protección de Cultivos* (Crop Protection Department) of the *Instituto de Agricultura Sostenible* (IAS-CSIC) in Cordoba, Spain. Isolates were stored by covering cultures on plum extract agar with liquid paraffin and keeping them at 4°C in the dark. Active cultures of isolates were obtained on chlortetracycline-amended water agar (1 l of distilled water, 20 g of agar, 30 mg of chlortetracycline) and were further subcultured on Potato Dextrose Agar (PDA; Difco Laboratories, Detroit, USA). Cultures on PDA were grown for 7 days at 24°C in the dark.

Eight-month-old plants of olive cvs. Arbequina and Picual were used. Plants were obtained by micropropagation techniques and provided by Cotevisa (L'Alcudia, Valencia, Spain). Arbequina and Picual are olive cultivars grown extensively throughout Spain [34]. Picual plants were inoculated with both *V. dahliae* pathotypes, while Arbequina plants were inoculated with the D isolate only.

Inocula of *V. dahliae* were produced in an autoclaved cornmeal-sand (CMS) mixture in flasks incubated at 24±1°C in the dark for 6 weeks. Infested CMS substrate was mixed thoroughly with an autoclaved soil mixture (clay loam/sand/peat at 1∶1∶1 vol/vol/vol) at a rate of 1∶10 (wt/wt) to reach an inoculum density of approximately 4×10^4^ CFU/g of soil for each of the *V. dahliae* isolates. Non-infested CMS mixed with the autoclaved soil mixture at the same rate explained above served as control. Plants were grown in soil tanks (Frisol S.A., Córdoba, Spain) placed inside a walk-in growth chamber adjusted to 24±1°C, 40 to 70% relative humidity, and a 14-h photoperiod of fluorescent light at 360 µE m^−2^·s^−1^ for 3 months. Pots with soil and plant roots were set inside the soil tanks at a constant temperature of 16, 20, 24, 28 and 32°C, with a maximum variation of ±1°C for each of temperatures. Plants were watered daily as needed and fertilized weekly with 100 ml of Hoagland's nutrient solution [35].

The experiment consisted of a three-way factorial treatment design with pathotype of *V. dahliae*, olive cultivar, and soil temperature as factors. For each soil temperature there were ten and six replicated pots (one plant per pot) for inoculated and non-inoculated plants, respectively, in a completely randomized design. The full experiment was repeated twice.

### 2. Disease assessment

Incidence (0 =  plant showing no disease symptoms; 1 =  plant showing disease symptoms) and severity of symptoms were assessed at 2- to 3-day intervals throughout the duration of the experiment, 3 months after inoculation. Disease severity was assessed by visually observing foliar symptoms in each individual plant and rating them on a 0 to 4 scale according to the percentage of foliage with disease symptoms, where 0 = 0%, 1 = 1 to 33%, 2 = 34 to 66%, 3 = 67 to 100%, and 4 =  dead plant [33]. Symptoms cause by the ND pathotype consisted on a dieback of twigs and branches where leaves turned light-brown, rolled back toward the abaxial side, dried up and remained attached to the symptomatic shoots; whereas, those caused by the D pathotype characterized by early drop of green, infected leaves that eventually gave rise to complete defoliation and necrosis of branches. Upon termination of the experiments, colonization of plant tissues by *V. dahliae* was determined in each plant by isolating the fungus on water agar amended with Aureomycin (1 l of distilled water, 20 g of agar, 30 mg of aureomycin). For each plant, six 5-mm-long stem pieces representative of the lower, middle and upper parts were thoroughly washed under running tap water for 30 min. The surface of the samples was disinfested in 0.5% NaClO for 1.5 min; next, the samples were rinsed with sterile water, plated onto the medium, and incubated at 24°C in the dark for 7 days [11], [33]. Colonies of *V. dahliae* were identified by microscopic observation of verticillate conidiophores and formation of microsclerotia. Data from the stem isolations of the pathogen were used to calculate the intensity of stem vascular colonization, determined as the percentage of stem pieces from which the pathogen was isolated.

### 3. Disease, stress, plant growth-related parameters, and data analyses

#### 3.1. Relationship between disease development and parameters associated with disease progress curves

Disease progress curves were obtained from accumulated disease severity scores over time in days from the date of inoculation. The nonlinear form of the Gompertz model was evaluated for goodness of fit to disease severity progress data using nonlinear regression analyses. In the Gompertz equation, DS(t)  =  *K* exp[*–B* exp(*–r* t)], where DS  =  disease severity, *K*  =  asymptote parameter, *B*  =  constant of integration, *r*  =  relative rate of disease increase, and t  =  time of disease assessment in days after inoculation.

To further assess disease development, four additional variables associated with disease progression were explored. These variables included (i) the incubation period (IP), established as the time in days to first symptoms or its reciprocal (IP_R_  =  1/IP); (ii) the final disease incidence and severity assessed at the end of the experiment; (iii) the standardized area under the disease severity progress curve (SAUDPC), calculated using the trapezoidal integration method standardized by duration of disease development in days [36]; (iv) the intrinsic rate of disease increase (*rho*) parameter estimates of the Gompertz model fitted to the disease severity progress data; and (v) the intensity of stem vascular colonization determined as the percentage of stem pieces from which the pathogen was isolated.

#### 3.2. Leaf-level stress-related parameters

In addition to disease-associated parameters, several stress-related parameters were measured in six leaves of each of ten (inoculated) and six (non inoculated, control) plants for each soil temperature and olive cultivar-*V. dahliae* pathotype combination. All measurements were taken at 2-week intervals, starting 20 days after inoculation until the end of the experiment, and comprised (i) leaf temperature; (ii) leaf chlorophyll fluorescence; (iii) leaf photochemical reflectance index; (iv) leaf chlorophyll content (through SPAD readings); and (v) ethylene production. For each stress-related parameter, average daily increase was determined as the standardized area under the parameter value progress curve over the observation period, calculated with the trapezoidal method. Such increase was used to assess the relationship between stress-related parameters and soil temperature and differences between experimental treatments in such parameters. Leaf temperature and steady-state chlorophyll fluorescence measurements were conducted with the PAM-2100 pulse-amplitude modulated fluorometer (Heinz Walz GMBH, Effeltrich, Germany). Steady-state chlorophyll fluorescence was also assessed separately with a second instrument designed to measure chlorophyll fluorescence (FluorPen, Photon System Instruments, Brno, Czech Republic). In addition, measurements of the leaf photochemical reflectance index [21], calculated as (R_570_–R_531_)/(R_570_+R_531_) [37], [38], [39], were obtained with a custom-designed instrument to measure the R531 and R570 spectral bands with a bandwidth of 10 nm (PlantPen, Photon System Instrument, Brno, Czech Republic). Leaf chlorophyll content was obtained with the SPAD-502 chlorophyll meter (Minolta Corp., Ramsey, NJ, USA). This instrument was used preferentially because of the strong relationship between its digital readings and real leaf chlorophyll content, as demonstrated by several authors (e.g., [40], [41]). Ethylene production was determined in the two youngest fully expanded leaves in the upper part of the plant. Leaves were separated from the stem and enclosed in 3-ml test tubes containing 50 µl tap water. Tubes were sealed with rubber caps and incubated in the dark at 24°C for 24 h. Before sampling, the test tubes were stirred to favor the diffusion of ethylene gas into the water. Gas samples were withdrawn from the incubation tubes with a 1 ml gas-tight syringe and assayed with a Hewlett Packard gas chromatograph (Model 5890A), as previously described [42].

#### 3.3. Plant growth-related parameters

At the end of the experiments, plants were removed from the soil and their roots were washed free of soil. We measured the weight of fresh roots and dry and fresh aerial plant parts as well as the length of stems and shoots of individual plants. For this latter parameter, we calculated a daily growth rate relative to the initial values.

#### 3.4. Relationship between disease, stress and plant growth-related parameters, and soil temperature

Three functions were used to describe the effects of soil temperature on disease, stress, and plant growth-related parameters for the different olive cultivar-pathogen pathotype combinations. We used the following reverse sigmoid function to determine the relationship between disease incidence, disease severity, and intensity of stem vascular colonization:
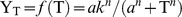
where Y_T_ is the response of disease-related parameters to soil temperature (T), *a* determines the maximum asymptote, and 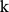 is the half maximum parameter.

For the remaining disease-related parameters and leaf temperature measurements, we used the modified beta function [43]:
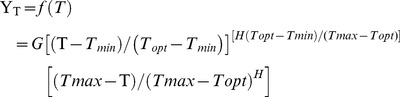
where Y_T_ is the response of disease-related parameters to soil temperature, and *T_max_* and *T_min_* are 36 and 8°C, respectively, which are known maximum and minimum temperatures for growth of *V. dahliae* isolates [16], [18], [19]. The shape parameter (*H*) determines the soil temperature range near the optimal soil temperature (*T_opt_*) at which the response values are close to the maximum response (*G*). For the remaining parameters, we used a Type I combined exponential and power model:



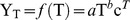
where Y_T_ is the response of the measured variable to soil temperature, and *a, b* and *c* are parameters that closely interact to control the shape of the curve [44].

All regression analyses were conducted using the Marquardt nonlinear least-squares iterative procedure for nonlinear models (NLIN) of SAS software (version 9.3; SAS Institute, Cary, NC, USA). The coefficient of determination (*R^2^*), mean square error, standard errors associated with the parameter estimates, confident intervals of predicted values, and pattern of standardized residuals plotted against either predicted values or the response variable were used to evaluate the appropriateness of models to describe the data [45].

#### 3.5. Relationships between experimental treatments and disease, stress, and plant growth-related parameters

The overall response of experimental treatment combinations to disease, stress, and plant growth-related parameters was first explored by cluster analyses. To establish functional groups of correlated experimental treatments, agglomerative clustering based on the Spearman correlation matrix was performed using the Ward clustering method [46]. The optimal number of clusters was estimated on the basis of the average silhouette width according to the Mantel statistic. The number of clusters in which the within-group mean intensity of the link between the objects (i.e., experimental treatments) and their groups was highest (i.e., with the largest average silhouette width) indicated the optimal cluster number. A dendrogram was then produced representing the treatment groups identified [46]. A heat map was developed to visualize the values of the different treatments and parameters used in the analysis. All cluster analysis calculations were performed using R software, version 3.0.2 (R Foundation for Statistical Computing, http://www.R-project.org/) with the *cluster* [47], *gplots* [48] and *vegan* [49] packages.

#### 3.6. Relationship between disease severity classes and stress-related parameters

Two approaches were used to assess the ability to discriminate among disease severity classes and stress-related parameters: logistic regression models and classification trees. Logistic models are direct probability models that are stated in terms of the probability of occurrence of an event (i.e., disease severity class) under a given set of conditions (i.e., stress parameters) [50]. In this study, a multinomial logistic regression model was fitted to each stress parameter as an independent-explanatory variable and disease severity class as the dependent-response variable, using healthy plants as the reference category. Multinomial models with ordinal responses are an extension of standard (i.e., binary) logistic regression to regression with three or more ordered categories [51]. To assess the statistical significance of each independent variable, each model was compared to the null model using a likelihood ratio test. The proportion of the variance explained by each model was evaluated using the maximum rescaled *R* ^2^ determination coefficient, and classification accuracy. This was done by using the LOGISTIC procedure of SAS software. To assess the combined effects of all stress-related variables, a multiple logistic regression model was fitted using the stepwise procedure. The developed model was externally validated by partitioning of individuals into two samples: the training sample containing 80% the data of each severity class selected at random and the testing or validation sample with the remaining 20%. The logistic model was fitted using the training sample and externally validated by using the testing sample to assess its classification accuracy.

Classification trees were used to determine the thresholds of stress parameters that discriminated between disease severity classes. The decision tree was obtained by recursive data partitioning, thereby splitting the data set into increasingly smaller subsets based on the predictive variables. The optimal tree was determined using the minimal estimate of cross-validated prediction error for different numbers of splits [52]. Residual mean deviance and misclassification error rates were used as a measure of goodness of fit of the selected tree. The analysis was conducted using the *rpart* package [53] in the R environment. The selected tree was validated by dividing the full data set into two parts and testing for classification accuracy, as described above for the logistic regression analyses.

## Results

### 1. Verticillium wilt development

#### Treatment effects

**Figure 1:**
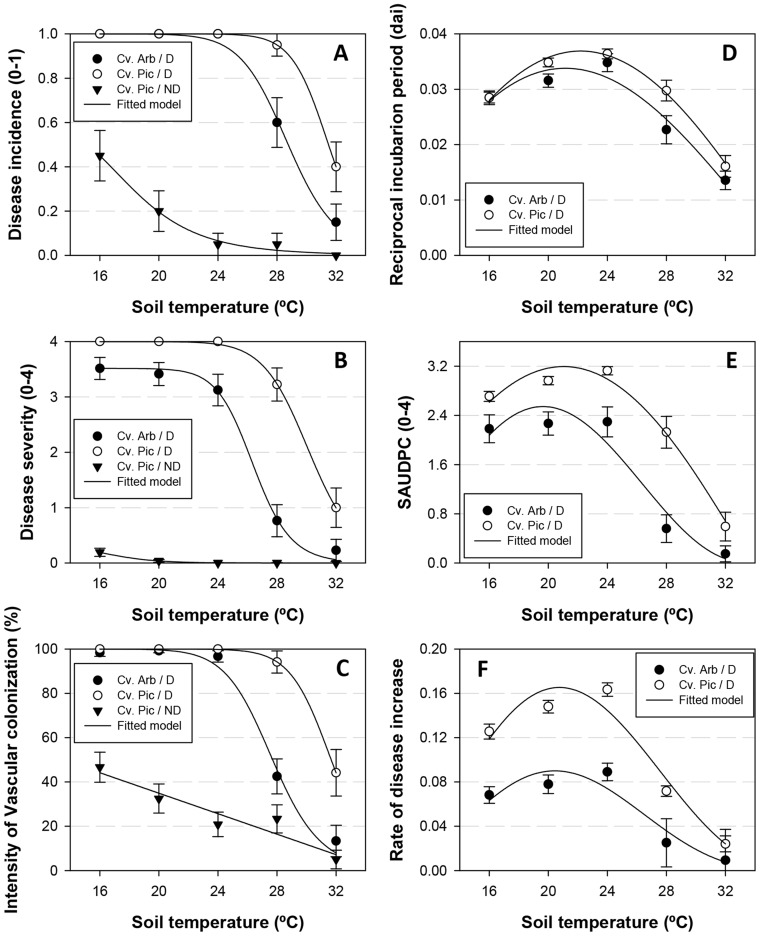
Relationship between Verticillium wilt-related parameters and soil temperature. Relationship between Verticillium wilt-related parameters and soil temperature in olive cvs. Arbequina (Arb) and Picual (Pic) grown in soil infested with the defoliating (D) or non-defoliating (ND) pathotype of *Verticillium dahliae*. **A.** Final disease incidence; **B.** Overall disease severity (0–4 scale: 0 = no symptoms, 4 = dead plant); **C.** Intensity of stem vascular colonization determined at the end of the experiment by isolation in growth media; **D.** Reciprocal incubation period (time to initial symptoms); **E.** Standardized area under the disease severity progress curve (SAUDPC); **F.** Rate of disease increase parameter of the Gompertz function fitted to disease progress data. Each point represents the mean of data from two repeated experiments, each comprising 10 pots with one plant per pot. Vertical bars represent the standard error of the mean. Lines represent the predicted model calculated with a reverse sigmoid function (left panels) or a Beta function (right panels).

Soil temperature, olive cultivar, and pathotype of *V. dahliae* were found to influence the development of Verticillium wilt in olive. Plants grown in soil infested with the D pathotype showed typical symptoms of the defoliating syndrome in the full range of soil temperatures tested, irrespective of the olive cultivar. Disease incidence, disease severity, and intensity of stem vascular colonization decreased with increasing soil temperature according to a reverse sigmoid model with asymptotic optimal values in the range of 16 to 28°C and 16 to 24°C for the interaction of the D pathotype with cvs. Picual and Arbequina, respectively (Fig. 1A–C). In these two cultivars, no significant differences (*P*≥0.05) existed between temperatures within the optimal range regarding levels of disease incidence or intensity of stem vascular colonization caused by the D pathotype (Fig. 1A–B). At 32°C, the three disease parameters (i.e., disease incidence, disease severity, and intensity of stem vascular colonization) decreased markedly in both cultivars, reaching 40.0%, 1.00 (on a 0–4 scale), and 44.2% in cv. Picual and 15%, 0.23, and 13.3% in cv. Arbequina, respectively (Fig. 1A–C). Disease was scarce in cv. Picual plants grown in soil infested with the ND pathotype and incubated at 16 to 28°C, and no symptoms were observed at 32°C (Fig. 1A–C). In this combination, disease incidence and intensity of stem vascular colonization was highest at 16°C (i.e., 46.7 and 45%, respectively) and decreased steadily with increasing soil temperature to 5% of stem vascular colonization at 32°C (Fig. 1C).

**Figure 2:**
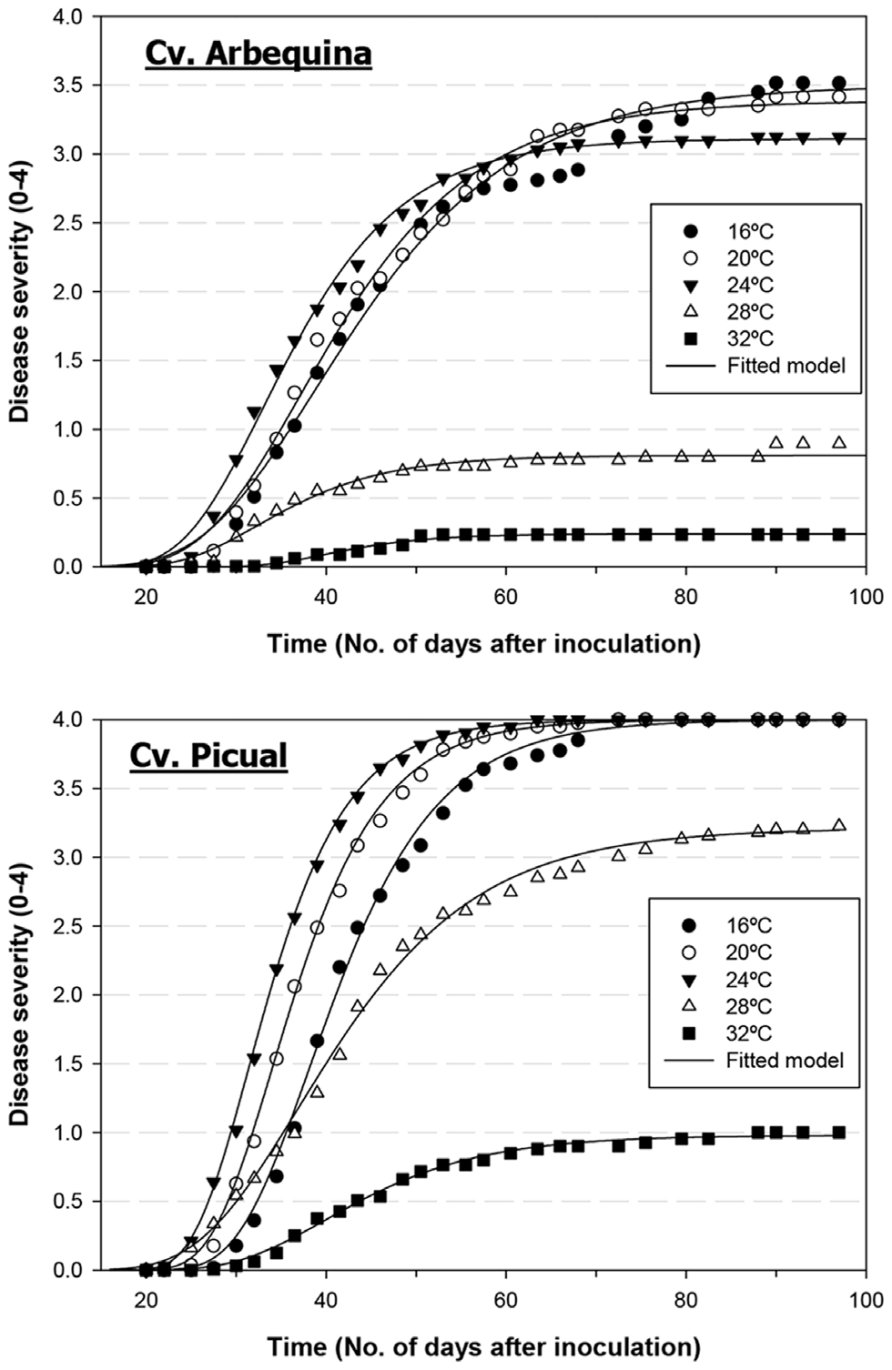
Verticillium wilt disease progress at different soil temperatures. Verticillium wilt disease progression in olive cvs. Arbequina and Picual grown in soil infested with the defoliating pathotype of *Verticillium dahliae* and incubated at different soil temperatures. Each point represents the mean disease severity (0–4 scale: 0 healthy, 4 = dead plant) of data from two repeated experiments, each comprising 10 pots with one plant per pot, at 2-to-3-day intervals. Solid lines represent the predicted disease progress curve calculated with the Gompertz function.

Time to symptom expression in olive plants was shortest at 24°C in both cultivars grown in soil infested with D *V. dahliae*. Symptoms started to develop 27 to 29 days after planting in cv. Picual and about 3 days later in cv. Arbequina; yet, symptom appearance was delayed the most at 20°C, followed by 16, 28, and 32°C (Fig. 1D). Disease development over time was adequately described by the Gompertz model (*R^2^*>0.97; RMSE <0.7631) for all cultivar-soil temperature combinations involving the D pathotype (Fig. 2). The increase in disease severity (DS) became asymptotic (i.e., DS>3 on a 0–4 scale) at all soil temperatures except for cv. Picual (DS = 1.0) at 32°C and cv. Arbequina at 28 and 32°C (DS = 0.77 and 0.23, respectively). Nevertheless, disease severity was always significantly greater (*P*<0.05) in cv. Picual than in cv. Arbequina regardless of soil temperature (Fig. 1C, Fig. 2).

The beta function adequately described the effects of soil temperature on the reciprocal of the incubation period, the standardized area under the disease severity progress curve (SAUDPC), and the intrinsic rate of disease progression (*rho* parameter of the Gompertz model fitted to temporal disease severity progress). Those three disease-related parameters increased within the range of 16 to 24°C and rapidly started to decrease at 28 and 32°C (Fig. 1D–F). Values of those parameters were significantly lower (*P*<0.05) in Arbequina plants compared to Picual plants infected with D *V. dahliae* at all soil temperatures, except for the incubation period at the extreme temperatures of 16 and 32°C, for which values were similar in the two cultivars (Fig. 1D–E).

### 2. Relationship between leaf-level measurements of stress-related parameters and soil temperature

**Figure 3:**
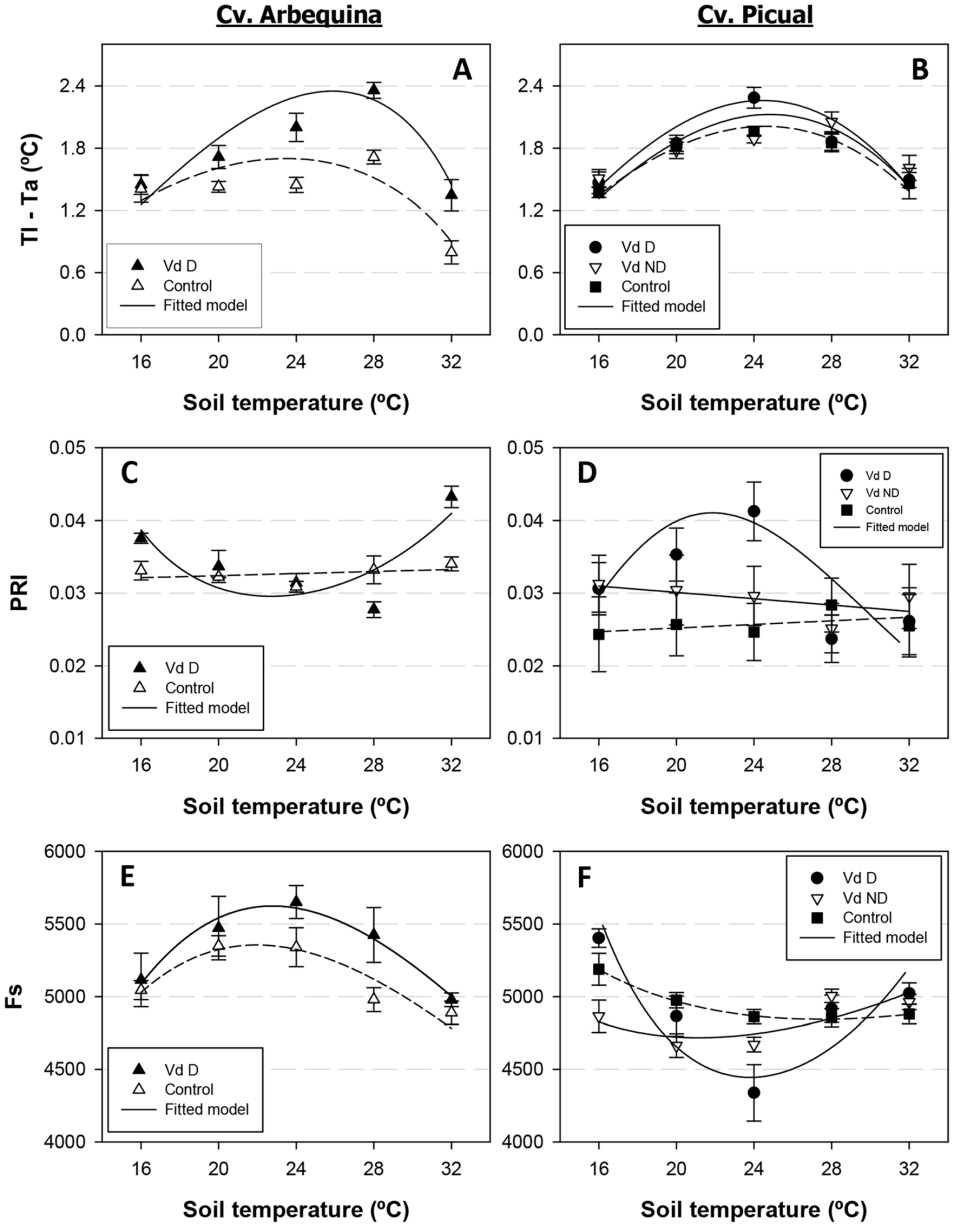
Relationship between stress-related parameters and soil temperature. Relationship between stress-related parameters and soil temperature in olive cvs. Arbequina (Arb) (left panels) and Picual (Pic) (right panels) grown in sterilized soil (control) or in soil infested with the defoliating (D) or the non-defoliating (ND) pathotype of *Verticillium dahliae*. **A.** Leaf temperature minus air temperature (Tl-Ta); **B.** Photochemical reflectance index (PRI); **C.** Steady-state chlorophyll fluorescence (Fs). Each point represents the mean of data from two repeated experiments, each comprising 6 pots with one plant per pot, at 2-week intervals. For each parameter, the average daily increase was calculated as the standardized area under the parameter value progress curve over the observation period. Vertical bars represent the standard error of the mean. Lines represent the predicted model calculated with a Beta function (leaf temperature minus air temperature) or a Type I combined exponential and power function.

**Figure 4:**
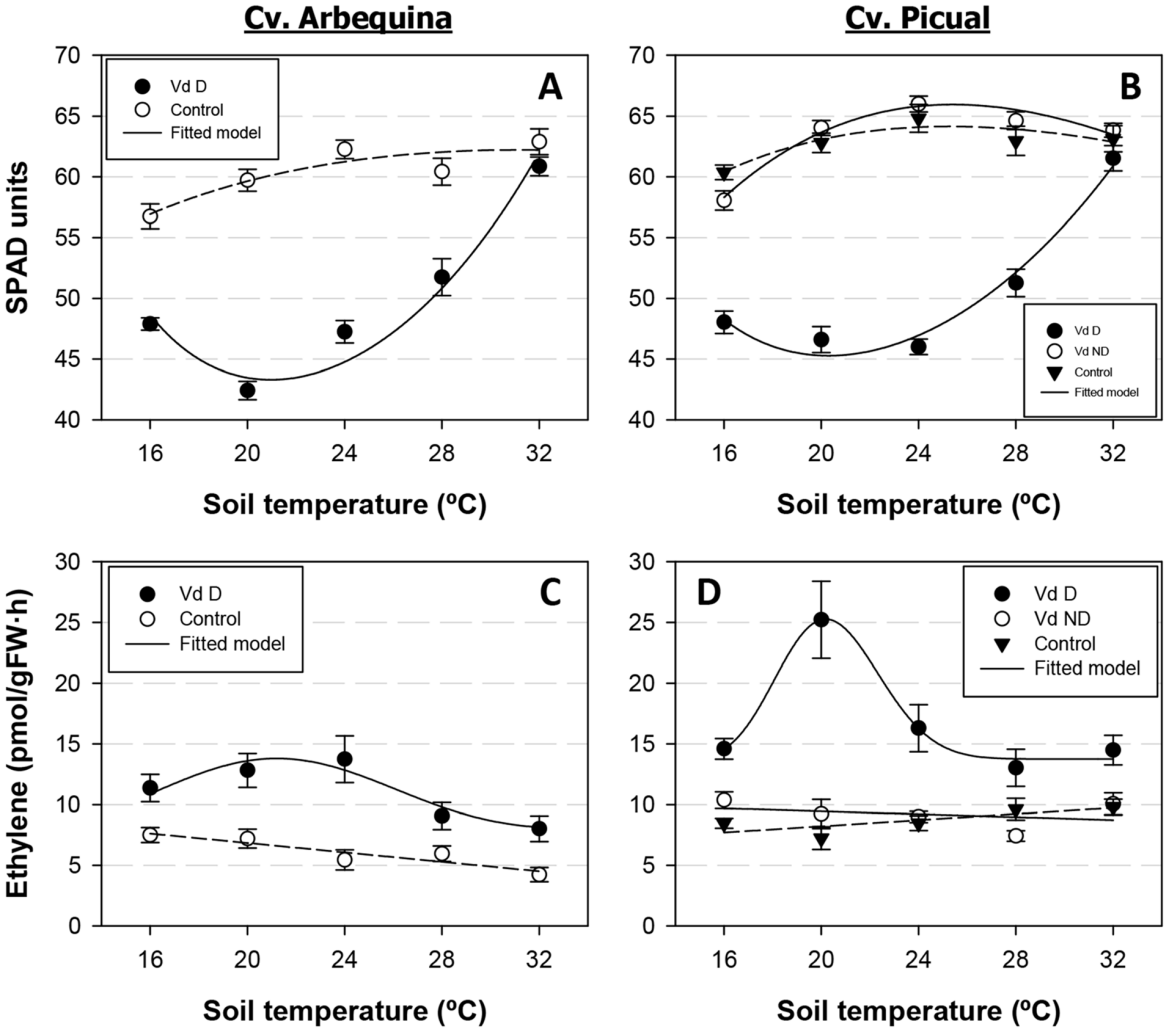
Relationship between stress-related parameters and soil temperature. Relationship between stress-related parameters and soil temperature in olive cvs. Arbequina (Arb) (left panels) and Picual (Pic) (right panels) grown in sterilized soil (control) or in soil infested with the defoliating (D) or the non-defoliating (ND) pathotype of *Verticillium dahliae*. **A.** Chlorophyll content (SPAD readings); **B.** Ethylene production. Each point represents the mean of data from two repeated experiments, each comprising six pots with one plant per pot, at 2-week intervals. For each parameter, the average daily increase was calculated as the standardized area under the parameter value progress curve over the observation period. Vertical bars represent the standard error of the mean. Lines represent the predicted model calculated with a Type I combined exponential and power exponential function.

Similarly to the disease-related parameters described above and for all olive cultivar-*V. dahliae* pathotype combinations, leaf temperature (estimated as the difference between leaf and mean air temperature) increased with soil temperature according to a beta model. Leaf temperature increased in the range of 16 to 28°C soil temperature and decreased at a soil temperature of 32°C (Fig. 3A). Leaf temperature in Arbequina plants infected with the D pathotype was 0.03 to 0.64°C higher than that of non-inoculated control plants at 20 to 32°C soil temperature (Fig. 3A). Conversely, there were minor differences in the leaf temperature of Picual plants infected with D or ND *V. dahliae*, although leaf temperature was higher in the most susceptible interaction – cv. Picual/D pathotype (1.47 to 2.29°C) −, decreased in plants infected with the ND pathotype (1.51 to 2.05°C), and was the lowest in control plants (Fig. 3B). Progression of the remaining four stress-related parameters with soil temperature was well described by a Type I combined exponential and power function. The photochemical reflectance index showed different relationships with soil temperature depending on the olive cultivar infected with D *V. dahliae* (Fig. 3C–D). Specifically, minimum values were measured in Arbequina plants grown at 24 and 28°C soil temperature, while the opposite occurred in Picual plants. Overall, however, both cultivars exhibited a similar range of PRI values: 0.03 to 0.04 (Fig. 3C–D). In the remaining three experimental treatment combinations, PRI values were not greatly modified by soil temperature. Values were highest in Arbequina control plants (in a range of 0.031 to 0.034), decreased to a range of 0.025 to 0.031 in the cv. Picual/ND pathotype interaction, and were lowest (0.024 to 0.026) in Picual control plants (Fig. 3C–D). Steady-state fluorescence also exhibited a different relationship with soil temperature depending on the olive cultivar. In cv. Arbequina, steady-state chlorophyll fluorescence values increased with higher soil temperatures ranging from 16 to 24°C and decreased at 28 and 32°C in both D-pathotype-infected and control plants. Yet, steady-state chlorophyll fluorescence values were lower in the control treatment group regardless of soil temperature (Fig. 3D–E). In cv. Picual, the highest steady-state chlorophyll fluorescence values were reached at the extreme 16 and 32°C soil temperatures. In this cultivar, at each soil temperature, steady-state chlorophyll fluorescence values were highest in control plants and tended to decrease in plants infected with *V. dahliae* regardless of the pathotype (Fig. 3D–E). Infection with the D pathotype had a strong effect on chlorophyll content estimated with SPAD readings. Specifically, in both olive cultivars, chlorophyll content was lowest in D *V. dahliae*-infected plants grown at 16 to 24°C (42.4 to 48.0 SPAD units), increased at 28°C (51.3 to 51.8 SPAD units), and reached the highest levels at 32°C (60.9 to 61.5 SPAD units). Chlorophyll content values exhibited minor differences between control plants of both cultivars and Picual plants infected with the ND pathotype at soil temperatures ranging from 20 to 32°C (59.7 to 66.0 SPAD units), but slightly lower values were recorded at 16°C (56.7 to 60.4 SPAD units) (Fig. 4A–B). Similarly, high ethylene production was detected mostly in olive plants infected with the D pathotype and was particularly higher in plants grown at 20 to 24°C, and in Picual plants (12.8 to 25.2 pmol g^−1^ root fresh weight h^−1^) compared to Arbequina plants. Ethylene production was always lower and almost constant irrespective of soil temperature for the remaining treatment combinations, ranging from 7.4 to 10.4 pmol g^−1^ root fresh weight h^−1^ in the cv. Picual/D pathotype combination and from 4.2 to 9.8 pmol g^−1^ root fresh weight h^−1^ in control plants of both cultivars (Fig. 4C–D).

**Figure 5:**
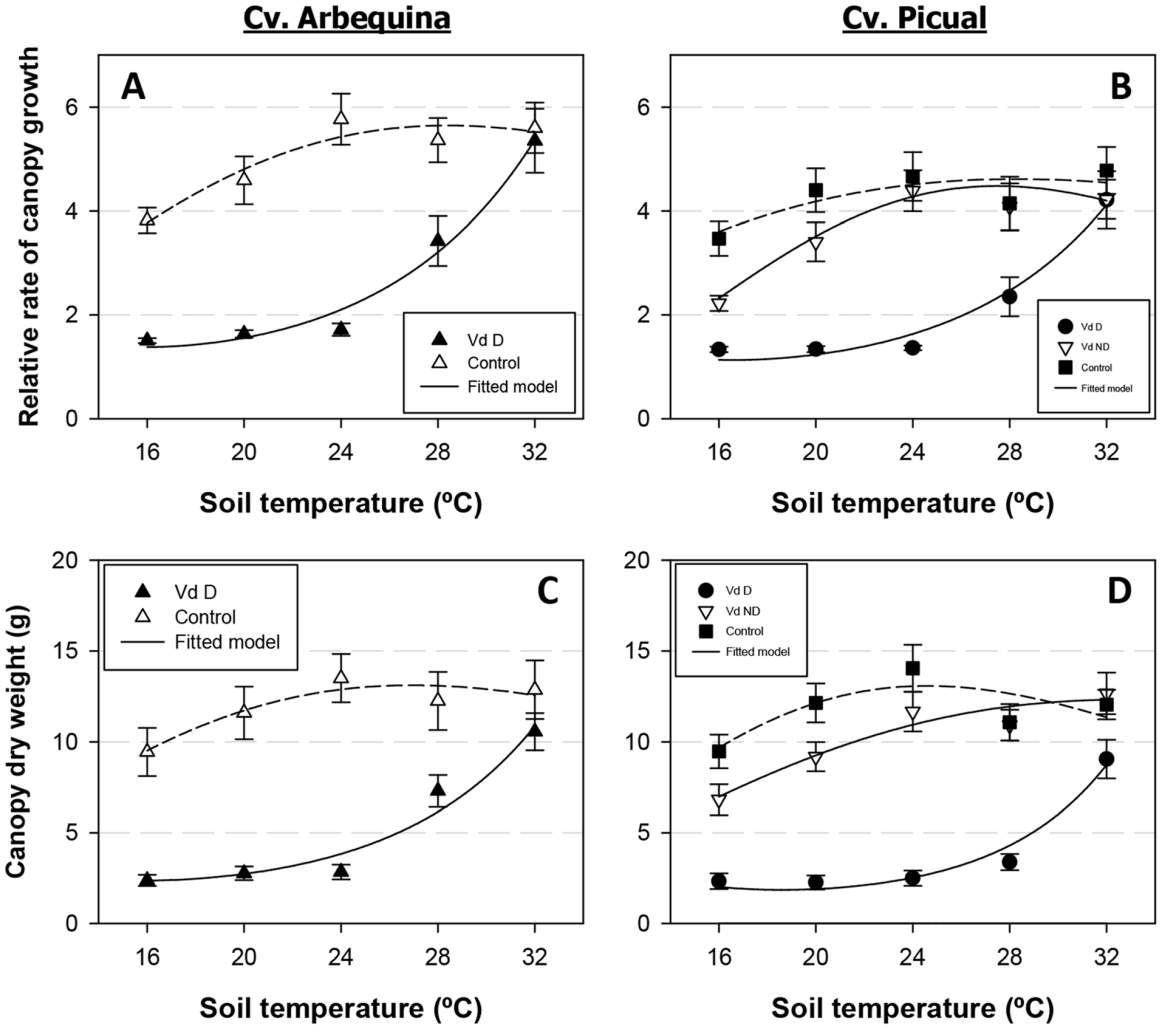
Relationship between plant growth-related parameters and soil temperature. Relationship between plant growth-related parameters and soil temperature in olive cvs. Arbequina (Arb) (left panels) and Picual (Pic) (right panels) grown in sterilized soil (control) or in soil infested with the defoliating (D) or the non-defoliating (ND) pathotype of *Verticillium dahliae*. **A.** Relative rate of canopy growth; **B.** Dry weight of the canopy. Each point represents the mean of data from two repeated experiments, each comprising six pots with one plant per pot, at the end of the experiments. Vertical bars represent the standard error of the mean. Lines represent the predicted model calculated with a Type I power exponential function.

Plant growth, estimated by the relative rate of canopy growth and dry canopy weight, was also strongly affected by the experimental treatments. The lowest values for both growth measures were observed in plants infected with the D pathotype of *V. dahliae* at a soil temperature ranging from 16 to 24°C. At these soil temperatures, based on measurements of canopy height at the beginning and the end of the experiments, Arbequina plants exhibited a 1.5 to 1.7 rate of canopy growth. The rate of canopy growth of Picual plants was 1.3 to 1.4 times higher than on Arbequina plants (Fig. 5A, C). At the same soil temperature levels, canopy dry weight values exhibited few differences, ranging from 2.4 to 2.8 g in cv. Arbequina and from 2.3 to 2.5 g in Picual plants; this parameter was about 75 to 82% lower than in non-infected control plants (Fig. 5B, D). At a soil temperature of 28°C, both growth parameters increased by 3.4 cm/day and 3.4 g/day in cv. Arbequina, and by 2.4 cm/day and 3.4 g/day in cv. Picual. At 32°C, maximum growth values were observed in all treatments and both cultivars. Plant growth of Picual plants infected with the ND pathotype was about 24 to 36% lower than that of control plants at a soil temperature of 16 and 20°C but minor differences were observed when plants grew at a soil temperature range of 24 to 32°C. In control treatments, plant growth tended to increase with the increase in soil temperatures but did so at a lower rate than in infected plants. Specifically, soil temperature reduced plant growth only in the lower soil temperature range of 16 and 20°C, and optimal maximum growth took place at 24 to 32°C (Fig. 5).

### 3. Relationships between experimental treatments and parameters related to disease, stress, and plant growth

**Figure 6:**
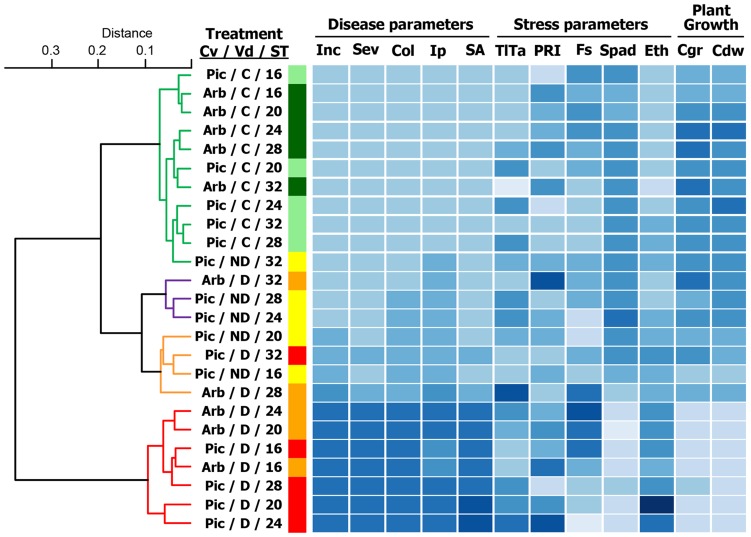
Dendrogram showing results of cluster analyses and heat map representation of disease, stress, and plant growth parameters. The 12 parameters selected for the heat map representation were related to Verticillium wilt reaction (5 parameters): Final disease incidence (Inc), Overall disease severity (Sev), Standardized area under disease severity progress curve (SA), Time to initial symptoms (Ip), and Intensity of stem vascular colonization (Col); related to stress (five parameters): Leaf temperature minus air temperature (TlTa), Steady-state chlorophyll fluorescence (Fs), Photochemical reflectance index (PRI), Chlorophyll content (Spad), and Ethylene production (Eth); and related to plant growth (two parameters): Relative rate of canopy growth (Cgr) and Dry canopy weight (Cdw). Agglomerative cluster analyses were performed based on the Spearman correlation matrix calculated from values of the different parameters using the Ward method. Cluster groupings of experimental treatment combinations represented in different colors were estimated on the basis of the average silhouette width according to the Mantel statistic. In the heat map, for each column, cells represent the relative value of each parameter for each experimental treatment combination of soil temperature, olive cultivar, and non-inoculated control and *Verticillium dahliae* pathotypes of the study from two repeated experiments.

To further analyze the interactions between *V. dahliae* pathotype, olive cultivar, and soil temperature, we performed a multivariate hierarchical cluster analysis, including all 12 parameters related to disease, stress, and growth as response variables. This analysis led to the creation of four functional groups – A to D – among the 25 experimental treatment combinations (Fig. 6). Group A included seven experimental treatments with a severe disease reaction, including plants infected with the highly virulent D pathotype grown at optimal soil temperature for Verticillium wilt development (i.e., 16 to 28°C for cv. Picual and of 16 to 24°C for cv. Arbequina). Overall, high values of disease severity-related parameters were associated with high levels of leaf temperature and ethylene production and low levels of chlorophyll content and growth-related parameters. In this group, Arbequina plants also exhibited high and low levels in PRI and steady-state chlorophyll fluorescence parameters, respectively; Picual plants exhibited the opposite pattern. Group B comprised four experimental treatments with a moderate disease reaction. It included Arbequina and Picual plants infected with the D pathotype and grown at 28°C and 32°C, respectively, and plants in the cv. Picual/ND pathotype combination grown at 16 or 20°C. This group of treatment combinations exhibited intermediate values in most of the parameters included in the study. Group C comprised three experimental treatments associated with a low level of disease. Specifically, plants in the cv. Arbequina/D pathotype combination grown at 32°C, and plants in the cv. Picual/ND pathotype combination grown at 24 or 28°C. The low Verticillium wilt development observed in treatment combinations within this group was associated with moderate values of stress and plant growth parameters. Group D comprised the remaining eleven treatments with healthy plants, including asymptomatic Picual plants inoculated with the ND pathotype grown at 32°C and all non-inoculated control plants of both cultivars at all soil temperature levels tested (Fig. 6).

### 4. Relationship between stress-related parameters and Verticillium wilt severity classes

**Figure 7:**
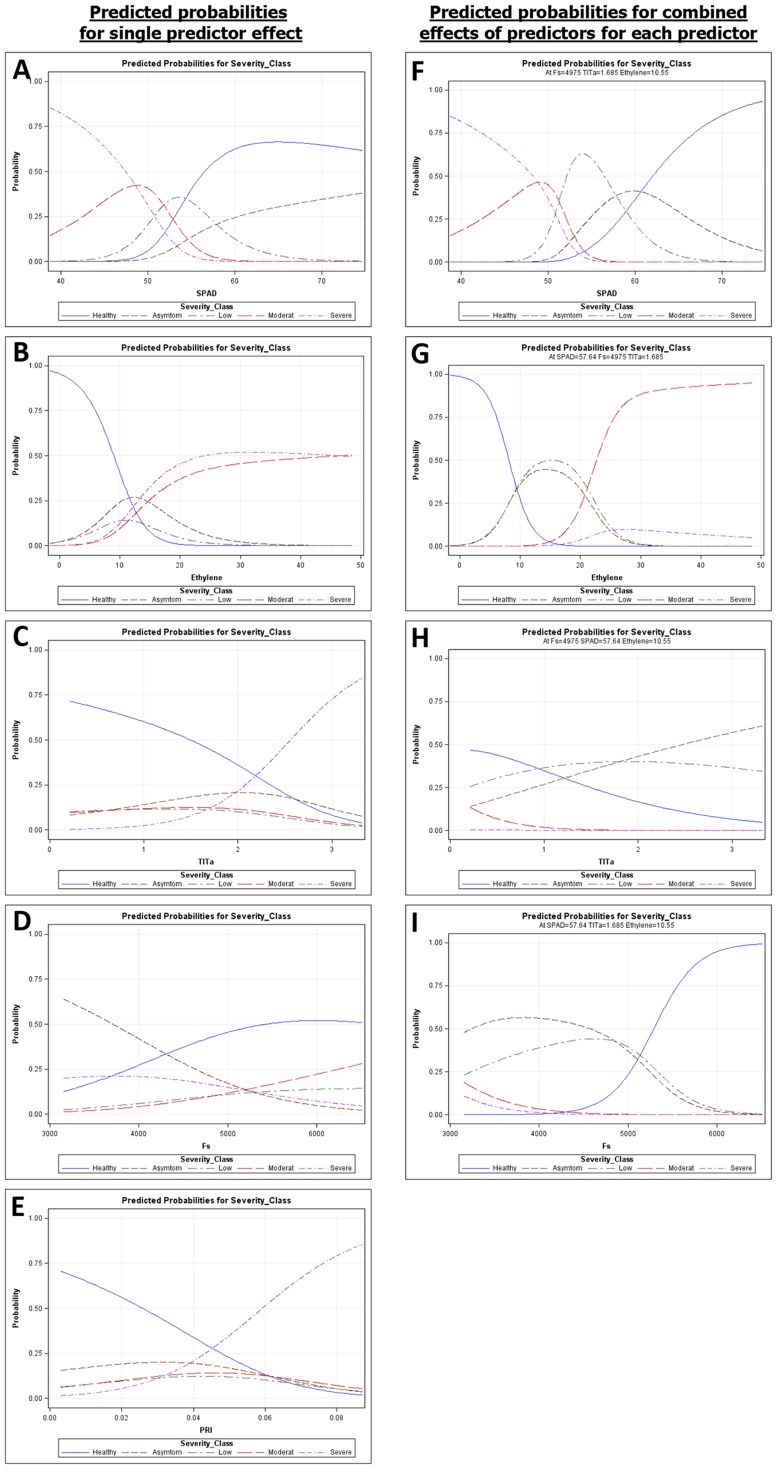
Predicted probabilities according to the multinomial logistic regression model with Verticillium wilt severity as the response variable and stress-related parameters as explanatory variables using healthy plants as the reference category. Left panels (**A to D**) represent the predicted probability distribution curves for each stress parameter fitted separately. Right panels (**E to I**) represent the predicted probability for each of the four stress parameters when the other three are fixed. **A, F.** Chlorophyll content (SPAD value); **B, G.** Ethylene production; **C, H.** Steady-state chlorophyll fluorescence (Fs); **D.** Photochemical reflectance index (PRI); **E, I.** Leaf temperature minus air temperature (Tl-Ta). Severity class indicates the severity of Verticillium wilt symptoms from Healthy control plants to Severe symptom development. Data include a training set of 188 plants selected at random from a total set of 235 plants in two repeated experiments comprising all experimental combinations of soil temperature, olive cultivars, and non-inoculated control and *Verticillium dahliae* pathotypes of the study.

A multinomial logistic regression analysis was performed to determine the relationship between stress-related parameters and Verticillium wilt severity classes. Logistic regression models fitted for each variable (Table 1, Fig. 7) exhibited significant differences between Verticillium wilt severity classes in all stress-related parameters. When fitted individually, chlorophyll content, ethylene production, and leaf temperature were the most explanatory parameters and had the highest correct classification rate (i.e., the Verticillium wilt severity class matched the assigned class with the highest probability) (Table 1, Fig. 7A–E). Although the PRI and steady-state chlorophyll fluorescence values were also statistically significant (*P*<0.011), their explanatory power and correct classification rate was lower (Table 1, Fig. 7E). In addition, we fitted a multiple logistic model using a stepwise procedure, selecting a model that included the four stress parameters with the highest explanatory power (i.e., chlorophyll content, ethylene production, leaf temperature, and steady-state chlorophyll fluorescence) but did not include the PRI. The model explained 98.75% of the total variability and correctly classified 73.40% of the cases (Table 1, Fig. 7F–I). Interestingly, 76 (91.6%) of the 83 healthy plants were correctly classified; of the remaining seven plants, three were classified as asymptomatic and four were classified as plants with only minor symptoms (*data not shown*), demonstrating the ability of the model to discriminate between healthy and diseased plants. Overall, in the selected model the probability of increase of Verticillium wilt symptom severity increased with growing leaf temperature and ethylene production, but the opposite occurred for chlorophyll content and steady-state chlorophyll fluorescence values. Figures 7F to I show the predicted probability distribution curves for the stress parameters included in the model keeping the remaining three stress parameters constant. The predicted probability distribution curves corresponding to Verticillium wilt severity classes from healthy to severely affected were distinct in all the parameters (Fig. 7). Moreover, chlorophyll content values showed distinct curves for all five severity classes (Fig. 7F), and ethylene production showed distinct probability curves for all Verticillium wilt severity classes except for asymptomatic and low symptom severity, which overlapped (Fig. 7G). Finally, leaf temperature (Fig. 7H) and steady-state chlorophyll fluorescence (Fig. 7I) showed distinct probability curves for healthy, asymptomatic, and low symptom severity classes, but overlapping curves for moderately or severely affected classes. The model was validated using a test data set containing 20% of the data of the original set that had a correct classification rate of 76.60% and explained 99.81% of the variation.

**Table 1:** Results of the multinomial logistic regression models fitted for each variable separately and the multivariate multinomial logistic regression model fitted with a stepwise method.

	LRT test^b^			
Predictor^a^	Chi-square	*P*> Chi-square	Max-rescaled *R^2^* b	Correct classification (%)^b^
SPAD	423.05	<0.0001	0.8964	59.57
Ethylene	122.30	<0.0001	0.4854	56.38
Tl-Ta	26.65	<0.0001	0.1358	49.47
Fs	16.78	0.0021	0.0869	47.74
PRI	13.17	0.0105	0.0888	46.28
SPAD, Ethylene, Tl-Ta, Fs	819.35	<0.0001	0.9875	73.40

a A multinomial logistic regression model was fitted to each of the stress parameters as an independent variable (predictor) and disease severity class as the dependent variable, using healthy plants as the reference category. To assess the combined effect of all stress-related variables, a multiple logistic regression model was fitted using the stepwise procedure. SPAD: Chlorophyll content; Ethylene: Ethylene production; Tl-Ta: Leaf temperature minus air temperature; Fs: Steady-state chlorophyll fluorescence.

b The likelihood ratio test (LRT), maximum rescaled *R^2^* determination coefficient, and correct classification rate were obtained when using the models for prediction.

### 5. Identification of stress-related parameter thresholds

**Figure 8:**
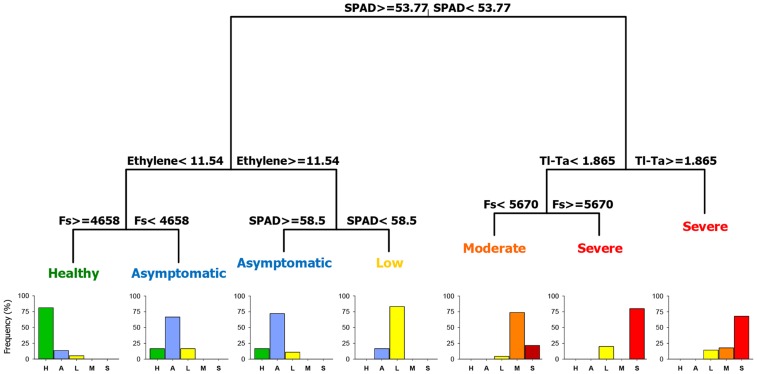
Classification tree to discriminate among Verticillium wilt severity classes based on stress-related parameters. Five stress-related parameters were used: Chlorophyll content (SPAD value), Ethylene production, Steady-state chlorophyll fluorescence (Fs), Leaf temperature minus air temperature (TlTa), and Photochemical reflectance index (PRI). Severity class indicates the severity of Verticillium wilt symptoms from Healthy control plants to Severe symptom development. For each terminal node the most prevalent Verticillium wilt severity class is indicated. The histogram for each terminal node represents the percentage of plants in each severity class (where H = Healthy, A = Asymptomatic, L = Low, M = Moderate and S = Severe symptoms). Data include a training set of 188 plants selected at random from a total set of 235 plants in two repeated experiments comprising all experimental combinations of soil temperature, olive cultivars, and non-inoculated control and *Verticillium dahliae* pathotypes of the study.

The optimal classification tree fitted to the data had seven terminal nodes and a discrimination ability of 76.6% (Fig. 8). Chlorophyll content (44.7%), ethylene production (37.3%), and steady-state chlorophyll fluorescence (30.4%) were the most important parameters in the construction of the classification tree. Leaf temperature and the PRI accounted for a much lower importance (i.e., 18.5 and 17.7%, respectively). Chlorophyll content was the main factor (i.e., first splitting stress parameter) that differentiated between plants in the healthy and low symptom severity class and those in moderate to severely affected classes with a chlorophyll content value <53.8 SPAD units. At the second level, ethylene and leaf temperature differentiated between both groups. Specifically, healthy plants were separated from asymptomatic plants by an ethylene production threshold of 11.5 pmol g^−1^ root fresh weight h^−1^. Plants below this threshold with a steady-state chlorophyll fluorescence >4658 were healthy, while plants with a lower fluorescence value were asymptomatic. In addition, plants with ethylene production above 11.5 pmol g^−1^ root fresh weight h^−1^ and a chlorophyll content value >58.5 SPAD units were asymptomatic and those with a lower chlorophyll content value exhibited mild symptoms. Moderate and severe Verticillium wilt classes separated by a chlorophyll content value <53.8 SPAD units were divided into two groups by leaf temperature: plants with a leaf temperature >1.9°C, which exhibited severe Verticillium wilt symptoms, and plants with a lower leaf temperature, which were in turn split in two new groups (i.e., plants with moderate Verticillium wilt symptoms and a steady-state chlorophyll fluorescence value <5670 and plants with severe Verticillium wilt symptoms and a steady-state chlorophyll fluorescence value above this threshold). The selected tree was validated using a test data set containing 20% of the data of the original set that was found to have a correct classification rate of 85.11%.

## Discussion

The development of Verticillium wilt in various crops has been related to environmental and soil conditions [16]. Soil temperature has been found to affect the development of diseases caused by *V. dahliae* in different crops [16]. However, no studies have explored the relationship between Verticillium wilt development and soil temperature in olive trees. The main objective of this study was to explore the relationship between soil temperature and Verticillium wilt symptom development, considering the potential influence of the virulence of *V. dahliae* pathotypes and the susceptibility of the host cultivar. We also measured several parameters at leaf level to assess the stress caused by the pathogen in olive plants at different soil temperatures and the relationship between such parameters and disease severity.

In our study, the most favorable soil temperature for Verticillium wilt development caused by the *V. dahliae* D pathotype was 24°C in both olive cultivars, showing the highest values in the five disease-related parameters measured (i.e., incubation period, final disease incidence and severity, standardized area under the disease severity progress curve, intrinsic rate of disease increase, and intensity of stem vascular colonization). At 16 and 20°C soil temperatures, high values of these parameters were also observed, although they were lower than those reached at 24°C. At 28 and 32°C, such disease parameters decreased dramatically, reaching the lowest values at 32°C. Our results are in close agreement with most studies on the effect of temperature on disease caused by *V. dahliae* in various host plants [16]. For example, studies conducted in cotton, tomato, and pepper infected by *V. dahliae* revealed that the optimal soil temperatures for *V. dahliae* growth were close to 20°C and temperatures higher than 28°C greatly reduced the development of the disease [16]. Temperature also interacts with the expression level of resistance of host cultivars. Specifically, severe disease symptoms caused by a highly virulent isolate can be modified by a temperature of 28°C and above to reach those of a moderately susceptible cotton cultivar [16], as occurred in our experiments with Picual plants infected with D *V. dahliae*. High temperatures delay germination of microsclerotia and therefore impair the ability of *V. dahliae* to penetrate the plant and cause disease [54], [55]. Values reached by disease-related parameters were higher in cv. Picual than in cv. Arbequina, as previously found by López-Escudero et al. [13], who reported that cv. Picual was more susceptible than cv. Arbequina to the D pathotype. Moreover, stress-related parameters showed the highest differences between D*-V. dahliae*-infected plants and control plants at 24°C. The reason for this was probably that 24°C was the most favorable soil temperature for Verticillium wilt development and consequently that plants at this soil temperature suffered the highest level of stress. Leaf temperature was higher in infected plants than in control plants, and the lowest differences them were recorded at 16 and 32°C soil temperatures. The higher leaf temperatures observed in infected plants than in control plants are consistent with the results of similar studies in oilseed rape plants infected by *V. dahliae* [31] and other soil-borne pathogens [26]–[31]. In potato, infection with *V. dahliae* has been found to cause lower stomatal conductance, lower transpiration, and therefore higher leaf temperature [56]. In sunflower plants, however, Sadras et al. [57] did not observe changes in leaf temperature or stomatal conductance due to infection with *V. dahliae*.

The photochemical reflectance index was higher in infected plants than in control plants in the cv. Picual/D pathotype combination; by contrast, this only occurred at a soil temperature of 16 and 32°C in the cv. Arbequina/D pathotype combination. These results are consistent with those obtained by Suárez et al. [37], [38] in trees subjected to water stress. Leaf chlorophyll fluorescence measurements estimated from steady-state chlorophyll fluorescence showed lower values in D pathotype infected plants of cv. Picual; in the cv. Arbequina/D pathotype combination, however, steady-state chlorophyll fluorescence values were higher in infected plants. The opposite trends observed in the two cultivars for the photochemical reflectance index and steady state fluorescence to soil temperature showed on Fig. 4C to F could be due to the differential response of both olive cultivars to infection by *V. dahliae* and soil temperature. Specifically, maximum disease severity and symptom expression is reached when Picual plants are grown at 20 to 28°C, while at these same soil temperatures disease severity and symptom expression on Arbequina plants are moderate or low. This is also supported by the minor differences showed between cultivars for both stress parameters at extreme soil temperatures (i.e., 16 and 32°C) where disease symptoms are low. The decrease in steady-state chlorophyll fluorescence in cv. Picual/D pathotype infected plants could be also expected according to previous results obtained with trees under water stress [39], [58]. Depression in photosynthetic activity mainly due to drought in plants inoculated with wilting fungi has been described in several pathosystems, including potato infected with *V. dahliae* [56], tomato infected with *Verticillium albo-atrum* [59], and *Quercus ilex* infected with *Cryphonectria parasitica* [60]. Chlorophyll content in leaves estimated by SPAD readings was inversely correlated with disease severity, as previously found in potato plants infected with *V. dahliae* [61]. Similarly, SPAD values related to chlorophyll content levels were able to indicate a reduction in photosynthetic activity in tomato plants [25] and grapevines [24] under water stress.

Ethylene is a plant hormone that acts as a signaling molecule in basal plant defense responses [62] and is known to increase rapidly upon *V. albo-atrum* infection [63]. In our study, ethylene production in leaf petioles was greater in infected plants of susceptible cv. Picual than in those of moderately susceptible cv. Arbequina. This agrees with the results of Birem et al. [64], who reported higher levels of ethylene in cv. Picual than in resistant cv. Frantoio plants due to infection by D *V. dahliae.* These findings are also consistent with those of Pegg and Cronshaw [63], who reported a significant production of ethylene in internodes of a susceptible cultivar, but low or no ethylene production in a resistant tomato cultivar following infection with *V. albo-atrum.*

The most favorable soil temperature for Verticillium wilt development of the *V. dahliae* ND pathotype in cv. Picual was 16°C. From that temperature upwards, values of disease incidence and severity and intensity of stem vascular colonization progressively decreased. These values were much lower than those reached by the D pathotype, as previously found by López-Escudero et al. [13], who reported that cv. Picual was more susceptible to the D pathotype than to the ND pathotype. In agreement with such findings, the greatest differences in stress-related parameters between ND pathotype infected plants and control plants were found at 16°C and decreased with rising soil temperatures. These differences were lower than those obtained in the D pathotype, confirming that the lower Verticillium wilt development in plants infected with the ND pathotype is related to a lower stress level. In plants infected with the ND pathotype, however, chlorophyll content and ethylene production showed similar values to those of control plants, revealing that photosynthesis and ethylene production were not affected by pathogen infection. In consequence, plant growth parameters of ND-pathotype-infected plants showed almost no differences when compared to those of control plants.

According to our results, the optimal soil temperature for D pathotype infection was in a range of 16 to 24°C in cv. Picual plants and 20 to 24°C in cv. Arbequina plants. The optimal soil temperature for ND pathotype development was in a range of 16 to 20°C. Differences found in the optimal temperature range for disease development for both *V. dahliae* pathotypes are in agreement with the optimal mycelial growth for isolates of the D and ND pathotypes. Indeed, in *V. dahliae* cotton isolates from southern Spain, Bejarano-Alcázar et al. [18] estimated that the optimal temperature for in vitro growth (over a 21 to 30°C range) was 24 to 27°C for isolates of the D pathotype compared to 21 to 24°C for the ND isolates. In addition, the lower temperature optimum for the ND pathotype may explain why this *V. dahliae* pathotype tends to be geographically restricted to cooler areas of southern Spain such as Granada and Huelva provinces, whereas the D pathotype is present in most olive growing areas but is particularly prevalent in the warmer areas of the Guadalquivir Valley [5].

The relationship between stress-related parameters and Verticillium wilt severity classes was determined by a multinomial logistic regression analysis and a tree classification. According to the multinomial regression models fitted for each parameter separately, significant differences were found among Verticillium wilt severity classes in all stress-related parameters. The parameters with the highest explanatory power were chlorophyll content, ethylene production, leaf temperature, and steady-state chlorophyll fluorescence. Predicted probability distribution curves corresponding to chlorophyll content values were different for all Verticillium wilt severity classes, which made it possible to discriminate between each disease severity level, even at early stages of *V. dahliae* infection. However, ethylene production showed similar probability curves for asymptomatic and initial-low symptom development classes, while leaf temperature and steady-state chlorophyll fluorescence were similar in moderately and severely affected plants. In fact, ethylene production of plants at early stages of Verticillium wilt development did not differ significantly from that of plants in the asymptomatic class, while leaf temperature and steady-state chlorophyll fluorescence showed significant differences between plants in such classes. Based on the results of the multinomial regression models fitted for each parameter separately, we fitted a multiple logistic regression model including the four best stress parameters: chlorophyll content, ethylene production, leaf temperature, and steady-state chlorophyll fluorescence. The model explained 98.75% of the variance and correctly classified 73.40% of the cases. The optimal classification tree had a similar discrimination ability (76.6%), revealing that chlorophyll content, ethylene production, steady-state chlorophyll fluorescence, and leaf temperature were the most important parameters. As shown by the logistic regression analysis, chlorophyll content was the main factor that differentiated between asymptomatic and low Verticillium wilt severity classes, while ethylene production did not distinguish between them. Nevertheless, leaf temperature and steady-state chlorophyll fluorescence differentiated between moderate and severe Verticillium wilt classes; by contrast, in the logistic regression analysis, these two parameters distinguished between all Verticillium wilt severity classes except moderate and severe classes. The PRI was the parameter with the lowest explanatory power and classification rate in both classification methods. These results obtained at leaf level under controlled conditions confirmed those obtained at leaf and canopy levels under field conditions by Calderón et al. [32], who proved the potential for early detection of *V. dahliae* infection and discrimination among Verticillium wilt severity levels in olive crops using thermal, multispectral, and hyperspectral imagery acquired with an unmanned aerial vehicle. In that study, temperature and chlorophyll fluorescence were identified as the best indicators to detect Verticillium wilt at initial stages of disease development, while the photochemical reflectance and chlorophyll indices proved to be good indicators to detect moderate and severe Verticillium wilt severity classes under field conditions.

In conclusion, the optimal soil temperatures for D and ND pathotype development were 20–24°C and 16–20°C, respectively, with a drastic reduction of Verticillium wilt symptom development at soil temperatures higher than 28°C. Cv. Picual plants were more susceptible to the D than to the ND pathotype and were more affected by the D pathotype than Arbequina plants. Stress-related parameters were able to detect the effects of *V. dahliae* infection and colonization on water flow that eventually cause water stress effects. Results demonstrated that leaf temperature, physiological indices (i.e., photochemical reflectance, steady-state chlorophyll fluorescence, and chlorophyll content), and ethylene production are related to physiological stress caused by Verticillium wilt. Chlorophyll content, steady-state chlorophyll fluorescence, and leaf temperature were identified as the best indicators to detect Verticillium wilt at early stages of disease development, while ethylene production and the photochemical reflectance index were good indicators to detect Verticillium wilt at advanced stages. In addition, chlorophyll content was the parameter with the highest explanatory power and correct classification rate in the classification models used in this study, followed by ethylene production, steady-state chlorophyll fluorescence, and leaf temperature. These results will be useful to better understand the differential geographic distribution of *V. dahliae* pathotypes in southern Spain found by Jiménez-Díaz et al. [5] and to assess the potential effect of climate change on the development of Verticillium wilt of olive under different future climate change scenarios.

## References

[1] Jiménez-Díaz RM, Cirulli M, Bubici G, Jiménez-Gasco LM, Antoniou PP, et al (2012) Verticillium wilt, a major threat to olive production: Current status and future prospects for its management. Plant Dis 96: 304–329. 10.1094/PDIS-06-11-0496 30727142

[2] Tsror L (Lahkim) (2011) Review: Epidemiology and control of Verticillium wilt on olive. Israel J Plant Sci 59: 59–69.

[3] Levin AG, Lavee S, Tsror L (2003) Epidemiology of *Verticillium dahliae* on olive (cv. Picual) and its effects on yield under saline conditions. Plant Pathol 52: 212–218.

[4] Ruggieri G (1946) A new disease of olive. L'Italia Agricola 83: 369–372.

[5] Jiménez-Díaz RM, Olivares-García C, Landa BB, Jiménez-Gasco MM, Navas-Cortés JA (2011) Region-wide analysis of genetic diversity in *Verticillium dahliae* populations infecting olive in southern Spain and agricultural factors influencing the distribution and prevalence of vegetative compatibility groups and pathotypes. Phytopathology 101: 304–315. 20942654 10.1094/PHYTO-07-10-0176

[6] Villalobos FJ, Testi L, Hidalgo J, Pastor M, Orgaz F (2006) Modelling potential growth and yield of olive (*Olea europaea* L.) canopies. Eur J Agron 24: 296–303.

[7] Wilhelm S (1955) Longevity of the Verticillium wilt fungus in the laboratory and field. Phytopathology 45: 180–181.

[8] Schreiber LR, Green RJ Jr (1963) Effect of root exudates on germination of conidia and microsc1erotia of *Verticillium albo-atrum* inhibited by the soil fungistatic principle. Phytopathology 53: 260–264.

[9] Talboys PW (1962) Systemic movement of some vascular pathogens. Trans Br Mycol Soc 45: 280–281.

[10] Ayres PG (1978) Water relations of diseased plants. In: Kozlowski TT, editor. Water Deficits and Plant Growth, vol. 5. London, United Kingdom: Academic Press. pp. 1–60.

[11] Navas-Cortés JA, Landa BB, Mercado-Blanco J, Trapero-Casas JL, Rodríguez-Jurado D, et al (2008) Spatiotemporal analysis of spread of *Verticillium dahliae* pathotypes within a high tree density olive orchard in southern Spain. Phytopathology 98: 167–180. 18943193 10.1094/PHYTO-98-2-0167

[12] Jiménez-Díaz RM, Tjamos EC, Cirulli M (1998) Verticillium wilt of major tree hosts: Olive. In: Hiemstra JA, Harris DC, editors. A Compendium of Verticillium Wilt in Tree Species. Wageningen, The Netherlands: Ponsen and Looijen. pp. 13–16.

[13] López-Escudero FJ, del Río C, Caballero JM, Blanco-López MA (2004) Evaluation of olive cultivars for resistance to *Verticillium dahliae* . Eur J Plant Pathol 110: 79–85.

[14] López-Escudero FJ, Blanco-López MA (2007) Relationship between the inoculum density of *Verticillium dahliae* and the progress of Verticillium wilt of olive. Plant Dis 91: 1372–1378. 10.1094/PDIS-91-11-1372 30780740

[15] DeVay JE, Forrester LL, Garber RH, Butterfield EJ (1974) Characteristics and concentration of propagules of *Verticillium dahliae* in air-dried field soils in relation to the prevalence of Verticillium wilt in cotton. Phytopathology 64: 22–29.

[16] Pegg GF, Brady BL (2002) Verticillium Wilts, Wallingford: CABI Publishing. 432 p.

[17] McKeen CD (1943) A study of some factors affecting the pathogenicity of *Verticillium albo-atrum* R. & B. Can J Res. 21: 95–117.

[18] Bejarano-Alcázar J, Blanco-López MA, Melero-Vara JM, Jiménez-Díaz RM (1996) Etiology, importance, and distribution of Verticillium wilt of cotton in southern Spain. Plant Dis 80: 1233–1238.

[19] Xu F, Yang L, Zhang J, Guo X, Zhang X, et al (2012) Effect of temperature on conidial germination, mycelial growth and aggressiveness of the defoliating and nondefoliating pathotypes of *Verticillium dahliae* from cotton in China. Phytoparasitica 40: 319–327.

[20] Jackson RD, Pinter PJ Jr (1981) Detection of water stress in wheat by measurement of reflected solar and emitted thermal IR radiation. In: Spectral signatures of objects in remote sensing. Versalles, France: Institut National de la Reserche Agronomique. pp. 399–406.

[21] Gamon JA, Peñuelas J, Field CB (1992) A narrow-wave band spectral index that tracks diurnal changes in photosynthetic efficiency. Remote Sens Environ 41: 35–44.

[22] Thenot F, Méthy M, Winkel T (2002) The photochemical reflectance index (PRI) as a water-stress index. Int J Remote Sens 23: 5135–5139.

[23] Papageorgiu G (1975) Chlorophyll fluorescence; an intrinsic probe of photosynthesis. In: Govindjee, editor. Bioenergetics of Photosynthesis. New York, USA: Academic Press. pp. 319–371.

[24] Flexas J, Briantais JM, Cerovic Z, Medrano H, Moya I (2000) Steady-state and maximum chlorophyll fluorescence responses to water stress in grapevine leaves: A new remote sensing system. Remote Sens Environ 73: 282–297.

[25] Fanizza G, Ricciardi L, Bagnulo C (1991) Leaf greenness measurements to evaluate water stressed genotypes in *Vitis vinifera* . Euphytica 55: 27–31.

[26] Hayat S, Hasan SA, Fariduddin Q, Ahmad A (2008) Growth of tomato (*Lycopersicon esculentum*) in response to salicylic acid under water stress. J Plant Interact 3: 297–304.

[27] Pinter PJ, Stanghellini ME, Reginato RJ, Idso SB, Jenkins AD, et al (1979) Remote detection of biological stresses in plants with infrared thermometry. Science 205: 585–587. 17729682 10.1126/science.205.4406.585

[28] Tu JC, Tan CS (1985) Infrared thermometry for determination of root rot severity in bean. Phytopathology 75: 840–844.

[29] Mengistu A, Tachibana H, Epstein AH, Bidne KG, Hatfield JD (1987) Use of leaf temperature to measure the effect of brown stem rot and soil moisture stress and its relation to yields of soybeans. Plant Dis 71: 632–634.

[30] Nilsson HE (1991) Hand-held radiometry and IR-thermography of plant diseases in field plot experiments. Int J Remote Sens 12: 545–557.

[31] Nilsson HE (1995) Remote sensing and image analysis in plant pathology. Annu Rev Phytopathol 15: 489–527. 10.1146/annurev.py.33.090195.002421 18999971

[32] Calderón R, Navas-Cortés JA, Lucena C, Zarco-Tejada PJ (2013) High-resolution airborne hyperspectral and thermal imagery for early detection of *Verticillium* wilt of olive using fluorescence, temperature and narrow-band spectral indices. Remote Sens Environ 139: 231–245.

[33] Mercado-Blanco J, Rodríguez-Jurado D, Parrilla-Araujo S, Jiménez-Díaz RM (2003) Simultaneous detection of the defoliating and nondefoliating *Verticillium dahliae* pathotypes in infected olive plants by duplex, nested polymerase chain reaction. Plant Dis 87: 1487–149. 10.1094/PDIS.2003.87.12.1487 30812391

[34] Barranco D, Fernández-Escobar R, Rallo L (2004) El cultivo del olivo (The olive tree crop), 5th edition. Madrid, Spain: Ediciones Mundi-Prensa. 800 p.

[35] Hoagland DR, Arnon DI (1950) The water culture method for growing plants without soil. California Agricultural Experiment Station, Circular No. 347.

[36] Simko I, Piepho H-P (2011) The area under the disease progress stairs: calculation, advantage, and application. Phytopathology 102: 381–389. 10.1094/PHYTO-07-11-0216 22122266

[37] Suárez L, Zarco-Tejada PJ, Sepulcre-Cantó G, Pérez-Priego O, Miller JR, et al (2008) Assessing canopy PRI for water stress detection with diurnal airborne imagery. Remote Sens Environ 112: 560–575.

[38] Suárez L, Zarco-Tejada PJ, Berni JAJ, González-Dugo V, Fereres E (2009) Modelling PRI for water stress detection using radiative transfer models. Remote Sens Environ 113: 730–740.

[39] Zarco-Tejada PJ, González-Dugo V, Berni JAJ (2012) Fluorescence, temperature and narrow-band indices acquired from a UAV for water stress detection using a hyperspectral imager and a thermal camera. Remote Sens Environ 117: 322–337.

[40] Yadava UL (1986) A rapid and nondestructive method to determine chlorophyll in intact leaves. HortScience 21: 1449–1450.

[41] Marquard RD, Tipton JL (1987) Relationship between extractable chlorophyll and an in situ method to estimate leaf greenness. HortScience 22: 1327.

[42] Romera FJ, Alcántara E, De la Guardia MD (1999) Ethylene production by Fe-deficient roots and its involvement in the regulation of Fe-deficiency stress responses by strategy I plants. Ann Bot 83: 51–55.

[43] Hau B (1988) Ein erweitertes analytisches modell für epidemien von pflanzenkrankheiten (An extended analytical model for epidemics of plant diseases). Giessen, Germany: Justus-Liebig-Universität. 183 p.

[44] Sit V, Poulin-Costello M (1994) Catalogue of curves for curve fitting. Biometrics Information Handbook Series, 4. Victoria, Canada: Forest Science Research Branch, Ministry of Forests. 116 p.

[45] Madden LV, Hughes G, van den Bosch F (2007) The Study of Plant Disease Epidemics. St. Paul, MN, USA: APS Press, The American Phytopathological Society. 421 p.

[46] Borcard D, Gillet F, Legendre P (2011) Numerical Ecology with R. New York, USA: Springer. 306 p.

[47] Maechler M, Rousseeuw P, Struyf A, Hubert M, Hornik K (2013) cluster: Cluster Analysis Basics and Extensions. R package version 1.14.4.

[48] Warnes GR, Bolker B, Bonebakker L, Gentleman R, Liaw WHA, et al.. (2013) gplots: Various R programming tools for plotting data. R package version 2.12.1.

[49] Oksanen J, Blanchet FG, Kindt R, Legendre P, Minchin PR, et al.. (2013) vegan: Community Ecology Package. R package version 2.0–10.

[50] Hosmer DW, Lemeshow S (2000) Applied Logistic Regression. 2nd ed. New York, USA: John-Wiley & Sons, Inc. 416 p.

[51] Agresti A (2007) An Introduction to Categorical Data Analysis 2nd ed. Hoboken, NJ, USA: John Wiley & Sons, Inc. 372 p.

[52] Everitt BS, Hothorn T (2010) A Handbook of Statistical Analysis Using R 2nd ed. Boca Raton, FL: Chapman and Hall/CRC Press. 348 p.

[53] Therneau T, Atkinson B, Ripley B (2013) rpart: Recursive Partitioning. R package version 4.1–4.

[54] Pullman GS, DeVay JE, Garber RH, Weinhold AR (1981) Soil solarization: effects on Verticillium wilt of cotton and soilborne populations of *Verticillium dahliae*, *Pythium* spp., *Rhizoctonia solani*, and *Thielaviopsis basicola* . Phytopathology 71: 954–959.

[55] Tjamos EC, Fravel DR (1995) Detrimental effects of sublethal heating and *Talaromyces flavus* on microsclerotia of *Verticillium dahliae* . Phytopathology 85: 388–392.

[56] Bowden RL, Rouse DL (1991) Effects of *Verticillium dahliae* on gas exchange of potato. Phytopathology 81: 293–301.

[57] Sadras VO, Quiroz F, Echarte L, Escande A, Pereyra VR (2000) Effect of *Verticillium dahliae* on photosynthesis, leaf expansion and senescence of field-grown sunflower. Ann Bot 86: 1007–1015.

[58] Zarco-Tejada PJ, Berni JAJ, Suárez L, Sepulcre-Cantó G, Morales F, et al (2009) Imaging chlorophyll fluorescence from an airborne narrow-band multispectral camera for vegetation stress detection. Remote Sens Environ 113: 1262–1275.

[59] Lorenzini G, Guidi L, Nali C, Ciompi S, Soldatini GF (1997) Photosynthetic response of tomato plants to vascular wilt diseases. Plant Sci 124: 143–152.

[60] El Omari B, Fleck I, Aranda X, Moret A, Nadal M (2001) Effect of fungal infection on leaf gas-exchange and chlorophyll fluorescence in *Quercus ilex* . Ann For Sci 58: 165–174.

[61] Gamliel A, Grinstein A, Peretz Y, Klein L, Nachmias A, et al (1997) Reduced dosage of methyl bromide for controlling Verticillium wilt of potato in experimental and commercial plots. Plant Dis 81: 469–474. 10.1094/PDIS.1997.81.5.469 30861924

[62] Fradin EF, Thomma BPHJ (2006) Physiology and molecular aspects of Verticillium wilt diseases caused by *V. dahliae* and *V. albo-atrum* . Mol Plant Pathol 7: 71–86. 20507429 10.1111/j.1364-3703.2006.00323.x

[63] Pegg GF, Cronshaw DK (1976) Ethylene production in tomato plants infected by *Verticillium albo-atrum* . Physiol Plant Pathol 8: 279–295.

[64] Birem F, Alcántara E, Blanco-López MA, López-Escudero FJ (2009) Physiologycal differences expressed by susceptible and resistant olive cultivars inoculated with *Verticillium dahliae*. In: 10th International Verticillium Symposium, Book of Abstracts. Corfu Island, Hellas, p. 71.

